# Barriers and Facilitators for a Collaborative International Physical Therapist Residency Program in Nairobi, Kenya

**DOI:** 10.3389/fpubh.2018.00266

**Published:** 2018-10-02

**Authors:** Shala Cunningham, Bini Litwin, Alicia Fernandez-Fernandez, Jennifer Canbek, Richard Jackson

**Affiliations:** ^1^Department of Physical Therapy, Radford University, Radford, VA, United States; ^2^Department of Physical Therapy, Nova Southeastern University, Fort Lauderdale, FL, United States; ^3^The Jackson Clinics, Middleburg, VA, United States

**Keywords:** residency education, physical therapy, barriers & facilitative factors, Kenya, phenomenology

## Abstract

**Introduction:** With the globalization of higher education through online training, opportunities exist for collaboration between institutions to promote ongoing advancement of healthcare professionals in resource-limited countries. The success of these programs is dependent on the ability of the program to meet the educational needs of the student and assist with implementation of the new information into practice. A post graduate residency program for physical therapists was introduced to Kenya to promote the development of the profession of physical therapy. This study sought to explore barriers that affected participation in the residency program, and how participants perceived the residency program fostered the use of new skills in the clinical environment, as well as the limitations they faced in applying the skills gained through the residency program in a clinical setting.

**Methods and Materials:** The participants in this study were in the third and fourth cohorts of the residency program (n = 27). One-on-one interviews were performed with the residents following completion of the program. A qualitative phenomenology research design was used to describe the manner in which the residency was experienced within the context of the environment in which the experience took place. Descriptions and narratives were obtained from the residents to provide a window into their lived experience.

**Results:** Four themes were discovered: (1) The ongoing challenge to balance often conflicting responsibilities: family, work and education, (2) A need to educate patients and colleagues on newly acquired skills to gain acceptance, (3) Success in the program requires reliance on support networks, and (4) Increased confidence gained in delivery of patient care.

**Discussion:** Although the residents faced obstacles for completing the residency and integrating newly acquired knowledge and skills into clinical practice, they were able to formulate strategies to meet these challenges. Understanding the barriers and facilitators that affect participants in international collaborative efforts may ultimately assist residency and other educational programs in designing new models of education, which will advance the physical therapy profession globally.

## Introduction

The increased availability of the Internet and online learning platforms allows students to access courses at university-level institutions across the globe. International education offerings vary from single online courses, to purely online degree programs, to hybrid curriculums that blend online learning with intermittent face-to-face time on campus to fulfill course requirements. Advances in technology and globalization of education programs provide students with opportunities to advance their profession and expand their skill set. However, the costs associated with these programs can be prohibitive to students in countries with limited resources wishing to participate in professional development.

According to the World Confederation for Physical Therapy (WCPT), education for entry-level therapists should include a minimum of 4 years of university level courses (1). There are currently three physical therapy education programs in Kenya. One institution offers a 3-year Diploma and two institutions provide a four-year Bachelor of Science degree (2). In addition to recommendations on entry-level education, the WCPT proposes that to promote development of the profession, physical therapists should be committed to pursuing educational opportunities following entry-level education (1). In 2013, the WCPT estimated there were 600 practicing physical therapists in the country (2).

Access to professional development opportunities following graduation is limited throughout the country of Kenya (3). The shortage of physical therapists with advanced degrees and specialty training has been a restrictive factor in the provision of educational opportunities following entry-level education (3). In addition, access to international education is often limited by high costs, which can prove prohibitive to physical therapists in resource-limited countries. Kenya is defined as a lower-middle income country by the World Bank, with an estimated gross national income (GNI) of $1,440 per capita in 2017 (4).

Opportunities for collaboration between international partners may help promote professional growth of participants without incurring excessive costs. One such opportunity lies in the development of a residency education program. A clinical residency program could provide a development opportunity for physical therapists following professional education, designed to advance the therapist's knowledge, skills, and clinical reasoning in a specific area of practice (5). The residency experience combines opportunities for ongoing mentoring with course work designed to provide a theoretical basis for advanced practice (5).

## Background and rationale

To promote skill advancement, clinical reasoning development, and use of current evidence in practice, the Jackson Clinics Foundation (Foundation) was invited in 2011 to meet with the administration of Kenya Medical Training College (KMTC) to assist in the development of a transitional Bachelor of Science degree for physiotherapists in Kenya who held a three-year diploma (6). A collaborative partnership was developed between the Foundation and KMTC. It was determined that a Bachelor of Science degree would be costly to administer and would not necessarily result in an increase in clinical skills of Kenyan physical therapists. It was then decided the most efficient and cost-effective method to increase clinical performance was through a Higher Diploma (residency program). The length of the program was determined by two factors. First, students would need to keep their clinical positions, requiring the program to be part time. Second, since the focus of the residency would be orthopedic, the discussion centered on how long it would take to teach each orthopedic module in depth including extensive hands-on labs. It was felt that 2-week long modules separated by 3 months would be optimal.

The 18-month post-graduate residency program was thus formed in Nairobi, Kenya, and is currently in its 6th year. The program focuses on providing advanced instruction for physical therapists without requiring a change in the entry-level educational structure in the country. The mission of the residency program is to graduate advanced orthopedic practitioners that can lead their communities and local profession in the advancement of clinical care and education (6).

## Description of the case

The residency program consists of six onsite modules and online education. The online didactic portion of the program includes background reading that includes the Clinical Practice Guidelines and Current Concepts in Orthopedics, 3rd edition (American Physical Therapy Association). Current research was provided by the instructors of each module to support evidence-based practice (6). Online materials were provided to the residents through a cloud sharing account. Materials were managed by an administrator in the United States to ensure residents received all materials prior to each onsite module.

The residency was developed using the United States model of residency education for physical therapists. There was a focus to ensure that the residency program, developed in a low-context culture, met the educational needs of the participants practicing in a high-context culture. Within a high-context culture, nonverbal communication is commonly based on an awareness of cultural norms. In a high-context educational system, face-to-face encounters with the instructor are used to explain course requirements and the written syllabus or handouts are often ignored (7). However, in a low-context culture, communication more often occurs explicitly. Written instructions and educational materials contain significant detail and are used as the primary resource. Learners from high-context cultures may have difficulty in online coursework, as communication styles differ and interaction is less personal (7). Students from high-context cultures have also been shown to be less open to participating in online discussions (7).

Due to specific societal and cultural orientation, students may be more amenable to learn in an environment that supports their cultural orientation to maximize learning (7). To make the program conductive to the students in Kenya, the structure of the residency was developed to include integrated onsite learning modules that allowed the Kenyan residents to interact directly with instructors and mentors throughout the program. Each onsite module consists of 10 days of education focused on skill development provided by physical therapy instructors from the United States. Instructors received an orientation to the culture of Kenya prior to traveling to the residency site. In addition to onsite modules and online resources, residents received clinical mentoring throughout the 18-month program. The mentorship focused on integrating the knowledge and skills learned during the residency program into clinical practice (6). Volunteer physical therapists from the United States provided the onsite mentoring in 2-week blocks. Mentoring occurred in Nairobi and surrounding areas within the resident's clinic. If a resident resided outside of Nairobi, arrangements a were made for commuting residents to provide care at a local facility. The success of the program has been dependent on its ability to meet the educational needs of the residents and assist with integration of newly acquired concepts into the residents' clinical practice.

To understand the residency experience from the residents' perspectives, individual interviews were conducted with the two resident cohorts in 2016. The interviews sought to explore barriers that affected participation in the residency program, how participants perceived the residency program fostered the use of new skills in the clinical environment, and the residents' perceptions of limitations for clinical application of the skills gained through the residency program.

## Methodological aspects

### Study approval

Approval for this study was received from the respective institutions. Informed consent was obtained prior to initiation of the study, and the rights and confidentiality of the participants were protected throughout the study.

### Subjects

This study utilized a sample of convenience of residents in the third and fourth cohorts of an orthopedic manual therapy residency program in Nairobi, Kenya.

## Methods

A qualitative phenomenology research design was used to describe the manner in which the residency was experienced within the context of the environment in which the experience took place (8). Descriptions and narratives were obtained from the residents to provide a window into their lived experience through one on one interviews conducted onsite by the primary investigator in September 2016. The primary investigator did not have access to the residents outside of the research study and was not associated with administration of the program. Participants were identified only by class cohort. Pseudonyms in the form of initials were assigned to participants. All interviews were recorded with participants' consent and transcribed by an independent transcriptionist to ensure accuracy. The transcripts and recordings were reviewed by the primary investigator prior to analysis.

The information obtained from semi-structured individual interviews was coded and general themes identified by the primary investigator. NVivo for Mac was utilized to arrange codes. Thick descriptions and narratives of the participants have been provided to inform the themes. To ensure credibility, all themes were confirmed through peer review by a member of the research study team with extensive qualitative research expertise. Member checks were performed with 10 of the residents.

Limitations in the qualitative assessment included potential bias in interpretation of the data. Potential bias of the researchers may include comparison of professional DPT students in the United States to residents in Kenya, in addition to bias as professional educators. To attempt to minimize bias, an investigator with no involvement in data collection or residency education performed an external audit of the data. The utilization of a sample of convenience and number of participants also limited generalizability of the qualitative results.

## Results

A total of Twenty-Seven residents participated in the study with a mean age of 33.3 (SD = 10.2) years. The participants included 15 males and 12 females with mean practice experience of 9.7 (SD = 9.8) years. All participants had completed a 3-year diploma in physical therapy. Their focus of practice included generalists working in both inpatient and outpatient environments (n = 10), outpatient orthopedics (n = 15) and outpatient pediatrics (n = 2).

Barriers and facilitators for participation in the residency program and integration of new skills in the clinic were relayed throughout the interviews. Residents discussed difficulty in balancing competing responsibilities during the residency program. With residents required to attend onsite modules in 2-week blocks, they needed 12 weeks of leave from home and work over the 18-month residency program. Female residents in particular discussed the difficulty in maintaining employment, meeting family responsibilities, and participating in the residency program. The residents that lived outside of Nairobi discussed the additional challenges of travel and the cost of staying in Nairobi to complete the onsite modules. In addition, residents relayed that it was challenging to get frequent and extended time off from work to attend the onsite modules. Additional barriers were described related to their ability to integrate new knowledge and skills into their respective clinical practices. The residents discussed the need to provide education to patients and colleagues to assist with acceptance of the change in practice. This facilitated the acceptance of the new skills.

To offset barriers, residents discussed support networks that served as facilitators and encouraged them throughout the residency program. Support came from family, employers, and the residency program itself. The residents discussed aspects of the residency that supported their success, including easy accessibility of online materials and the ability to communicate directly with a local administrator. As the residents were able to clinically apply newly acquired skills, their confidence in providing effective patient care increased, further motivating them to continue in the program.

## Theme 1: the on-going challenge to balance often conflicting responsibilities: family, work, and education

Residents with young children reported difficulty in managing family responsibilities and the time commitment required of the residency program. One resident, A.A., stated, “Being a family person, it's hard having your kids to juggle through school and the family. It's hard, but I thank God I was able to get through.” Another resident with a young child, N.K., similarly noted, “I have a small baby. My baby is just growing and sometimes leaving my baby for two weeks, for two weeks [sic], sometimes it's hard.”

In addition to the time commitment required for the residency program and the time away from home for the onsite modules, the sacrifice to take time off from work was noted. S.M. explained,

You miss work on such day because they tell you no we are not going to pay you and maybe that money was in your budget. So, this is some of the difficulties. Also paying, the paying [sic] back the thing that you lost at work; you have to work overtime, you miss your social life, your family life. So, you pay back heavily to come to class.

D.M. described a similar challenge, noting, “The biggest barrier we encountered these schedule [sic] for work and class. It's very difficult for your employer to understand that you need to come to class every cohort and you miss work on such day [sic].”

Residents also described difficulty maintaining productivity levels as they attempted to integrate new skills. Many residents also found the number of patients needing treatment often limited their ability to practice all assessment skills learned in the residency program. As explained by S.K.S.;

Most of the time we are challenged by the numbers. Although I see between ten and sixteen, I also have other duties to do in the process. So that makes it even more challenging to have adequate time with the patient to do that [sic] examination.

M.D. noted strategies to deal with the limitations. “Some things you do for the next, the next visit. You can't do everything all day. So, you're like okay, this is what this is, the presentation. What can I do?”

An added financial and time burden was an issue for the three residents that lived outside of Nairobi and had to travel to the onsite residency modules. O.N. traveled a considerable way to Nairobi and described what it meant to him to do so.

There be [sic] challenges maybe with finances there [sic] and being somebody that works outside the capital city. I started when I was um at the border of Kenya and Somalia and Sudan. I would come. It's over 1600 kilometers, so travel three days to be here.

## Theme 2: a need to educate patients and colleagues on newly acquired skills to gain acceptance

As the residents integrated newly acquired skills and knowledge into the clinic, they faced the challenge of gaining patients and colleagues acceptance about new techniques that meant a change in practice approach. Residents described the need to educate patients regarding manual treatment options and alternatives to modalities. J.O. explained, “Challenges are there especially when it comes to the patients. It was the patients who are used to, you know, hot packs, ultrasound and all that. Now you are coming to do something else.” T.D. confirmed this observation, explaining:

Okay, the problem. Because like if you get those patients who are used to this hot pack and want me to use this. I want to use manual therapy. So, some of them are not really ready to cooperate. They will do everything (manual therapy and exercise). They will still say, ‘I want hot pack.’

Residents discussed the need to use various strategies to encourage patients to accept new techniques. C.E. noted she focused on flexibility and compromise to gain the patient's acceptance of manual treatment as well as provide the modalities that patients expected, “Sometimes my patients could always want to see like [sic] at least a machine has to be used, but then I struggle to make sure I do manual therapy. Then give the machine to those that like them [sic].”

The residents discussed promoting a new approach to patient care by providing formal instruction and education to their colleagues, as well as serving as a consultant to their peers for difficult patients. This transference of knowledge provided colleagues with additional techniques for treatment. A.A. reported the opportunity to provide instruction to colleagues after each onsite module. “In our clinic, we have days where we do Continuous Medical Education. So, during the hours that we're given to do [sic], I come out there. I teach them what I learned from here and they are positive.”

Education provided to other disciplines was perceived as being able to improve patient outcomes beyond the immediate physical therapy community and to promote interprofessional teamwork as explained by J.N.

Does it really change? I was talking to my colleagues, not only physios but doctors, and I was telling them what I learned in clinical reasoning; the ICF, the manual skills, everything. How everything is good. It is increased [sic], not only when in physio condition, but in general health care conditions. You find that in most conditions, you often have to involve everyone.

W.S. further reported providing education through consultation, “I'm actually being consulted with what I am about, [sic] any condition about orthopedics and physiotherapy. Because it gets hard to them [sic], they refer to me. Call me and tell me, we are stuck here. How do you go about this?”

## Theme 3: success in the program requires reliance on support networks

The residents noted personal support networks, employer support, and facilitators within the residency program as factors that assisted their participation in the residency program. Support included financial support, emotional support, accessibility of residency materials, and encouragement from mentors from the United States. W.S., who traveled a long distance to Nairobi, described the benefit of receiving support from his family that enabled him to pursue the residency.

I got support from my family, very much. And family, including my sister, because I come like four hundred miles away. So, when I'm in Nairobi for the residency, I get support from my sister for upkeep for the period of the module.

In addition to financial support, one resident noted the help provided by her spouse in caring for their children. “What really helped, my husband helped me a lot. Supported me with the kids.”

Some residents received support from their employers that facilitated participation in the residency. Employer support was described as financial and flexible work assignments within practice areas. J.N. described how she “….got the financial support from the hospital I work. They paid for me. And I think to me, that was the best support you could ever get, allowing me to come.”

In addition to financial support, residents noted how their work setting facilitated their learning process by giving them the increased opportunity to practice newly acquired skills in the outpatient setting during the residency training. S.K.S. explained:

Yes, the institution allowed us to practice immediately when we started the program. Like me for instance, I used to work in the inpatients, but I was brought to the outpatient where several patients with musculoskeletal issues come to. So, I was able to daily practice [sic] what I learned from school.

J.N. also noted support from management as patient satisfaction improved, “The most important is the support from the management. The management actually they are very supportive as a program about getting compliments from my clients and even now trying to engage all of the hospital management to also embrace OMT.”

Residents noted the organization and accessibility of resources made available by the residency program helped facilitate participation and completion of the residency. All information necessary for the online modules was available in a cloud sharing account. Residents could download or print the materials while on campus. This negated the need to purchase textbooks or access the internet from community facilities. The residents noted that access to course materials before the onsite modules provided the opportunity for pre-reading and increased preparation for class. O.N., commented, “More so the information that is sent online. It makes it much easier for you to just study from where you are and then when you come, you at least [sic] you've internalized something and you get to flow well with the teachings.”

The role of the onsite administrator, who provided residents with a contact during and between modules, as well as coordination of modules, was seen as a strong contributor to the success of the residents' completing the program. Development of a consistent personal relationship between administrator and residents was important in promoting successful interaction between the residency instructors and the residency participants. The onsite administrator was crucial in ensuring consistent, ongoing communication and coordination of the modules. This was recognized as a strong support by O.N.

That the schedule has been kind of friendly with the arrangement of the leadership of the Mr. D. He was able to arrange well and then he communicates [sic] people in advance, so that makes it easier for you to plan yourselves to be able to attend. And then he has also been also good in terms of the payments for the school… So, I think that's what assisted in terms of attending.

Residents also discussed support provided by the mentors and instructors from the United States, recognizing the value of their encouragement and enthusiasm toward teaching. E.O. explained, “The lecturers they come prepared. They personally, they really encouraged me and when I was ever in doubt they always came and told me that I just didn't think simple. So, they really encouraged me.” This sentiment was reinforced by K.O. who noted, “All the facilitators have been very good and they've also made us come this far, encouraged us, and if we had questions they were ready to answer any questions that we had when we did not understand, the reading.”

## Theme 4: increased confidence gained in delivery of patient care

Residents found the information taught in the residency to be clinically applicable, and the utilization of patients as models in class reinforced how to apply that newly acquired knowledge. The ability to apply the new skills in the clinic led to the residents' perception of improved patient outcomes. As patient outcomes appeared to improve, residents gained confidence and motivation to continue to enhance their skills and knowledge. C.E. commented, “Actually the experience has made me, like I do not fear any of the condition or presentation of patients that has [sic] to my clinic. It has made me like, I am more brave, more able to assess any of the patients and able to treat any of the conditions in regards to musculoskeletal system.” This confidence aided communication with other medical providers. One resident, W.S., who traveled within Africa to provide PT services, noted how increased knowledge allowed him to discuss patient care confidently with others.

I would travel with a Kenya team sometimes, athletics team, but there, I would say, you see other stuff physios do and you compare with what you do.

You say wow, did I really do anything to help, but after doing this, I think I'm confident enough I can face anybody and I can discuss with my colleagues any condition.

## Discussion

Although the residents faced obstacles for completing the residency and integrating newly acquired knowledge and skills into clinical practice, they were able to formulate strategies to meet these challenges. Support from the residency was discussed by residents in relation to program planning to facilitate the integration of online teaching materials into a high-context culture. The residents noted the ability to clarify information provided in the online materials as they progressed through the program was highly valued. They also reported that the instructors willingness to answer questions until the entire cohort understood the concepts being presented was a great asset. This allowed the instructor to give a variety of examples of concepts and decreased the uncertainty of the information provided in online materials. Murray et al. (9) suggest that understanding the “why” of evidence-based practice improves the implementation of new approaches. The interaction and efforts of instructors to provide that “why” was described as a facilitating factor in gaining understanding of concepts and skills.

In addition to teaching concepts, all instructors and mentors from the United States were considered powerful motivators as they encouraged the residents to continue their participation in the program. Collectivism is considered a component of high-context cultures, where the community works together to learn skills and the success of the individual is regarded as a success for the community (7). The social network in which the high-context learner exists is extremely important as the learner navigates the online educational environment (10). If the social network is absent, the learner may feel abandoned (10). Collectivist beliefs may have extended beyond the program itself, as residents discussed the support networks that enabled them to be successful in the program, foremost family and to a somewhat lesser extent, their employers.

The residency program used, an online document sharing portal to provide resources to residents. The cloud sharing application provided a shared folder containing materials and resources for students to access electronically. The folder automatically synced each time the residents were connected to the Internet for onsite modules. This allowed the residents to access updated course materials and literature without having to download each file or having to become accustomed to an online learning management system.

Access to materials online has been considered an educational barrier in high-context cultures due to learners having difficulty accessing resources (7). Hofstede noted that the technology expectation in high-context cultures is easy familiarity with the learning portal and prescribed curricula (10). Despite this, the residents discussed online access as a positive aspect of the program. This may be because residents were instructed on how to download the cloud sharing application onto an electronic device during residency orientation. Since the cloud sharing account was organized by module and included the APTA resources, current literature, and instructor handouts for each session, residents were able to access multiple resources without the need for purchasing expensive textbooks or paper for printing.

In contrast to facilitating factors identified by the residents for participation in the residency, the female residents in particular noted difficulty maintaining a work-life balance while participating in the residency program. Work-life imbalance is experienced when participation in one role makes it difficult to participate in the other. This results in work and life duties that are incompatible (11). The concern over the ability to pursue higher education and to maintain time for family and friends has also been reported a barrier in the nursing profession (12). Morgenthaler noted lifestyle changes as a deterrent for nurses to pursue advanced degrees (12). This barrier has been noted not only in the United States, but in a study of woman engineers in Malaysia as well (13). Miller noted that balancing family and work is the most significant barrier in womens' attempts to advance (13).

The residents in this study reported increases in overall job satisfaction as patient satisfaction improved following residency education, despite a lack of change in salary or job promotion. This is different than perceptions of physical therapists in the US., where lack of career advancement through job promotions resulted in decreased overall job satisfaction (11). Similar to research on healthcare workers in the United States, the Kenyan physical therapists reported increased confidence with treatment decisions and care of complex patients following the residency. Improved confidence and self-esteem have been reported in nursing and dietary professions after attainment of an advanced degree post-professionally (14).

The Kenyan residents also discussed the need for patient education to develop a shared understanding of a treatment plan that included manual therapy. Patient education provided by the residents appeared to encourage patient willingness to be open to new techniques. McSweeney et al. discussed the need to examine the differences between the healthcare professional's explanatory model (EM) and the patient's EM before planning and providing treatment options (15). Once the healthcare professional and the patient share an EM, they can negotiate a treatment plan that meets the patient's expressed health needs. Residents discussed this negotiation as they incorporated modalities into the treatment plan with the new manual therapy techniques.

The residency's hybrid structure was able to meet the students learning needs within a high context culture while providing an affordable education experience. The residents noted several barriers for completing the residency program and incorporating new skills in the clinic, however, they were able to overcome these barriers and successfully advance their clinical practice. Understanding the barriers and facilitators that affect participants in international collaborative efforts may ultimately assist residency and other educational programs in designing new models of education which will advance the physical therapy profession globally.

## Lessons learned

In addition to developing an effective education program through collaboration with international partners, sustainability and independence need to be fostered to ensure continued promotion of the profession within the country. During the first two cohorts, three individuals were identified to participate in the onsite modules as teaching assistants. These individuals were considered by the Foundation and KMTC as those who would ensure sustainability of the program over time. In 2018, all of the courses have been taught by these three individuals, with mentors from the United States providing feedback and instruction on teaching style and module content. One webinar was arranged in which teaching methodology and teaching strategies were taught. In addition, the principles of developing effective presentations were reviewed.

In addition to the onsite modules and online courses, mentoring appeared to be a significant contributor to the integration of new skills into the clinic. Up to 150 h of mentoring is required in APTA credentialed residency programs (5). This level has been impossible to sustain in the Kenyan Residency due to the costs associated with the travel of mentors from the United States. It was determined that at least 50 h of mentoring was attainable. Initially, residency participants from outside the Nairobi area traveled to the city for mentoring by physical therapists from the United States, but this proved difficult for the residents. To decrease costs associated with mentoring, allow for extensive travel throughout the country, and provide consistent mentoring throughout the residency program, the Foundation has hired one of the leading teaching assistants to perform mentoring of students and graduates. This has proven very successful in both mentoring and recruitment of therapists to join the program.

The initial establishment of the residency program and close oversight by Foundation and US based volunteers over the initial 3 years, followed by a gradual progression toward a self-sustaining program through the training of graduates and strong support from an onsite administrator, has required frequent in-country visits by the United States administrative personnel. Dedication and collaboration of administration and Foundation volunteers has been fundamental to the stability and consistency of the program. Continual monitoring of the program's outcomes is necessary to define areas for improvement during the transition to an independent Kenyan run program. The long-term success of the program is perceived to be dependent on the transfer of responsibility and training to local physical therapists.

## Ethics statement

This study was carried out in accordance with the recommendations of the Kenya Medical Training College Ethics and Research Committee, the Institutional Review Board of Radford University, and the Institutional Review Board of Nova Southeastern University with written informed consent from all subjects. All subjects gave written informed consent in accordance with the Declaration of Helsinki. The protocol was approved by all three above committees.

## Author contributions

SC performed literature review, designed the methodology, performed interviews, and identified general themes within the data. BL assisted with study design, methodology, the generation of qualitative themes, and with revision and editing of the final document. RJ provided information on the development of the program, lessons learned, and future directions for the program. AF-F and JC provided guidance throughout the project, and assisted with revising and editing the final document.

### Conflict of interest statement

RJ is the founder of the residency program in Kenya. The remaining authors declare that the research was conducted in the absence of any commercial or financial relationships that could be construed as a potential conflict of interest.
